# Retraction: Endoplasmic reticulum Ca^2+^ release causes rieske iron-sulfur protein-mediated mitochondrial ROS generation in pulmonary artery smooth muscle cells

**DOI:** 10.1042/BSR-20192414_RET

**Published:** 2020-04-23

**Keywords:** Intracellular calcium, mitochondrial ROS, Ryanodine receptor

The authors of the following paper “Endoplasmic Reticulum Ca^2+^ Release Causes Rieske Iron-Sulfur Protein-mediated Mitochondrial ROS Generation in Pulmonary Artery Smooth Muscle Cells” (*Bioscience Reports* (2019) 39(12) https://doi.org/10.1042/BSR20192414) would like to retract their paper. They have provided the following justification for retraction:

The results in the published paper indicated that mitochondrial ROS generation was increased by hypoxia treatment (see Figure 4A). Inhibition of RyRs with tetracaine or genetic deletion of RyR2 attenuated hypoxia-induced ROS generation in mitochondria (see Figure 4A,B). However, the authors have been unable to repeat the results in subsequent experiments, and their latest data have shown that deprivation of RyR2 is not able to attenuate hypoxia-induced ROS generation in mitochondria.

**Figure 4:**
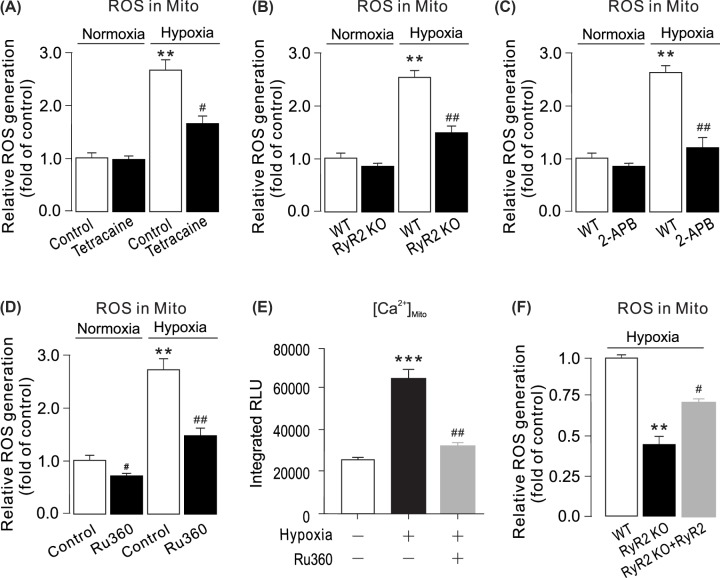
Inhibition or genetic deletion of RyR2 blocks hypoxic ROS production in mitochondria of PASMCs (**A**) PASMCs were pretreated with tetracaine (1 μM) (C) for 5 min followed by hypoxia for 5 min. Mitochondrial ROS generation were measured using a Mitochondrial ROS Detection Assay Kit. ** P < 0.01 compared with normoxia control group, and ##P < 0.01 compared with hypoxia control group. (**B**) PASMCs from WT and RyR2 KO mice were exposed to normoxia or hypoxia for 5 min. Mitochondrial ROS were measured by a Mitochondrial ROS Detection Assay Kit. Data were obtained from 3 separate experiments. **P < 0.01 compared with control, and #P < 0.05; ##P < 0.01 compared with WT group. (**C**) WT and RyR2 KO PASMCs were pretreated with 2-APB (20 μM) for 5 min followed by hypoxia for 5 min. Mitochondrial ROS generation were measured using a Mitochondrial ROS Detection Assay Kit. ** P < 0.01 compared with normoxia control group, and ##P < 0.01 compared with hypoxia control group group. (**D**) Cells were treated with Ru360 (1 μM) for 5 min, then exposed to caffeine (20 mM) for 5 min. mitochondrial ROS were measured by using a Mitochondrial ROS Detection Assay Kit. ** P < 0.01; *** P < 0.001 compared with normoxia control group, and ##P < 0.01 compared with hypoxia control group. [Ca^2+^]_mito_ was determined by mitochondria-targeted double-mutated aequorin (pcDNA3.1+/mit-2mutAEQ). Ru360 (1 μM) decreased hypoxia-caused [Ca^2+^]_mito_. (**E**) RyR2 KO PASMCs were transfected with RyR2 overexpression plasmids. All groups were treated with hypoxia for 5 min. Mitochondrial ROS generation were measured using a Mitochondrial ROS Detection Assay Kit. ** P < 0.01 compared with WT group, and #P < 0.05 compared with WT RyR2 KO group. (**F**) Mitochondrial ROS generation were measured using a Mitochondrial ROS Detection Assay Kit. **P < 0.01 compared with WT group, and #P < 0.05 compared with WT RyR2 KO group.

All authors agree to the retraction, and apologise for any misunderstanding caused by their results.

